# How Insertion of a Single Tryptophan in the N-Terminus of a Cecropin A-Melittin Hybrid Peptide Changes Its Antimicrobial and Biophysical Profile

**DOI:** 10.3390/membranes11010048

**Published:** 2021-01-12

**Authors:** Ana Rita Ferreira, Cátia Teixeira, Carla F. Sousa, Lucinda J. Bessa, Paula Gomes, Paula Gameiro

**Affiliations:** 1 Laboratório Associado para a Química Verde da Rede de Química e Tecnologia (LAQV-REQUIMTE), Departamento de Química e Bioquímica, Faculdade de Ciências, Universidade do Porto, 4169-007 Porto, Portugal; ritaferreira@fc.up.pt (A.R.F.); catia.teixeira@fc.up.pt (C.T.); cfilipams@gmail.com or lucinda.bessa@fc.up.pt (L.J.B.); pgomes@fc.up.pt (P.G.); 2 Helmholtz Institute for Pharmaceutical Sciences Campus E8 1, 66123 Saarbrücken, Germany

**Keywords:** antibiotic resistance, CAMP, cecropin-melittin peptides, tryptophan, cation-π interactions, large unilamellar vesicles, membrane-targeting activity

## Abstract

In the era of antibiotic resistance, there is an urgent need for efficient antibiotic therapies to fight bacterial infections. Cationic antimicrobial peptides (CAMP) are promising lead compounds given their membrane-targeted mechanism of action, and high affinity towards the anionic composition of bacterial membranes. We present a new CAMP, W-BP100, derived from the highly active BP100, holding an additional tryptophan at the N-terminus. W-BP100 showed a broader antibacterial activity, demonstrating a potent activity against Gram-positive strains. Revealing a high partition constant towards anionic over zwitterionic large unilamellar vesicles and inducing membrane saturation at a high peptide/lipid ratio, W-BP100 has a preferential location for hydrophobic environments. Contrary to BP100, almost no aggregation of anionic vesicles is observed around saturation conditions and at higher concentrations no aggregation is observed. With these results, it is possible to state that with the incorporation of a single tryptophan to the N-terminus, a highly active peptide was obtained due to the π–electron system of tryptophan, resulting in negatively charged clouds, that participate in cation–π interactions with lysine residues. Furthermore, we propose that W-BP100 action can be achieved by electrostatic interactions followed by peptide translocation.

## 1. Introduction

Antibiotic resistance is recognized by the World Health Organization (WHO) as a global public health threat. The overuse and misuse of antibiotics has led to the increase of multidrug-resistant (MDR) bacteria belonging to the “ESKAPE” pathogens group, comprising the strains *Enterococcus faecium*, *Staphylococcus aureus*, *Klebsiella pneumoniae*, *Acinetobacter baumannii*, *Pseudomonas aeruginosa*, and *Enterobacter* spp, which are listed in the 2017 WHO priority pathogen list, and are most commonly associated to nosocomial infections [1]. Few antibiotics have reached the market in the last two decades, and recently eight new antibacterial agents were approved, though eliciting few clinical benefits over existing treatments [2]. The loss of investment by large pharmaceutical companies led to the decline in the discovery of new antibiotics, so new antibacterial agents are urgently needed.

Cationic antimicrobial peptides (CAMP) are part of the innate immune system of many organisms including humans, bacteria, plants, insects, and amphibians [3,4]. They are a promising alternative to antibiotics since most of them target and permeabilize the bacterial membranes, making them less prone to elicit resistance as described for conventional antibiotics [5]. Additionally, they are short and amphiphilic molecules, with broad-spectrum activity, and selectivity against bacteria over eukaryotic cells. Both cationic and amphipathic properties of CAMP are key on their mechanism of action (MOA), promoting their interaction and incorporation into the negatively charged bacterial membranes by means of electrostatic and/or hydrophobic interactions [5]. Their attractive properties and antimicrobial activity against bacteria, viruses, and fungi have place CAMP in the research pipeline for the discovery of new antibacterial agents, with many being selected for phase II–IV of clinical trials, mostly for topical application [6]. 

Cecropin A-melittin hybrid peptides, such as BP100 (KKLFKKILKYL-NH_2_), are interesting candidates as new antibacterial agents, due to their potent antibacterial and membrane targeting activities. They result from the combination of two well-known naturally occurring peptides, cecropin A from the silk moth [7,8] and melittin from bee venom [9], aiming at shortening their length, while enhancing its therapeutic index and reducing production costs [10,11]. BP100 was firstly designed to fight phytopathogenic bacteria and fungi [12]. It is a widely studied amphipathic CAMP with high activity against susceptible and clinically relevant Gram-negative bacteria (minimum inhibitory concentration (MIC) values ranging from 2 to 5 µmol dm^−3^) [13,14], exhibiting low cytotoxicity and susceptibility to proteolytic degradation [12]. 

The substitution or incorporation of cationic/hydrophobic amino acid residues or hydrophobic moieties into CAMP sequences have been explored to yield peptide analogues with improved activity, stability, and selectivity/cytotoxicity against bacteria over mammalian cells [15,16]. In this connection, Torcato and co-workers were successful at improving the antibacterial activity of BP100 against Gram-positive bacteria by replacing lysine (Lys) and tyrosine (Tyr) amino acids for arginine (Arg) and tryptophan (Trp), respectively, not only potentiating hydrogen bond formation with the phosphate groups of phospholipids, but also increasing peptide’s hydrophobicity and the ability to deeply insert into the membrane of Gram-positive bacteria [15]. The amphipathic nature of CAMP and the balance between electrostatic and hydrophobic interactions with bacterial membranes are determinant for their membrane targeting activity, such is the case of cecropin–melittin hybrid peptides [10,13,17,18] or Trp-rich peptides [19,20,21]. Trp has a well-known high affinity for the interfacial region of biological membranes [22,23], potentiating the interaction of Trp-containing peptides with membrane surfaces [20,24]. 

In view of the above, in this work we produced and studied a novel cecropin A-melittin hybrid peptide, W-BP100, obtained through coupling of only one additional Trp to the N-terminus of BP100, i.e., possessing the amino acid sequence WKKLFKKILKYL-NH_2_. The synthesis, antibacterial activity and W-BP100 interactions with model membranes using steady-state fluorescence spectroscopy and dynamic light scattering (DLS) are herein reported and discussed. W-BP100 is not only active against Gram-negative bacteria but possess high activity against Gram-positive bacteria revealing a high partition constant towards anionic over zwitterionic large unilamellar vesicles (LUV), and a preferential location for hydrophobic environments. This finding is very important as it provides information for CAMP design, when a broader antibacterial activity is pursued, giving insights, at a molecular level, about the structural differences that can clarify CAMP action against Gram-positive and Gram-negative bacteria.

## 2. Materials and Methods

### 2.1. General Reagents

The lipids 1-palmitoyl-2-oleoyl-glycero-3-phosphocholine (POPC) and 1-palmitoyl-2-oleoyl-sn-glycero-3-phospho-(10-rac-glycerol) (POPG) were obtained from AVANTI Polar Lipids, USA. Chloroform (CHCl_3_, Reagent plus^®^, purity ≥ 99.8%), 4-(2-hydroxyethyl)piperazine-1-ethanesulfonic acid hemisodium salt (HEPES, purity ≥ 99%), sodium chloride (NaCl) and 5(6)-carboxyfluorescein (CF, purity ≥ 95%) were obtained from Sigma-Aldrich (Saint Louis, MO, USA). Unless otherwise stated, all solutions were prepared in 10 mmol dm^−3^ HEPES buffer containing 150 mmol dm^−3^ NaCl, using Milli-Q water, at pH 7.4, to mimic the physiological ionic strength [15].

### 2.2. Spectroscopic Measurements

Absorption spectra were carried out on a UV–vis–NIR (UV-3600) spectrophotometer (Shimadzu, Kyoto, Japan) equipped with a temperature controller (Shimadzu TCC-CONTROLLER). Spectra were recorded at 25 ± 0.1 °C, in 1 cm quartz cuvettes, with a slit width of 5 nm, in a wavelength range from 225–350 nm. Peptide concentrations with absorbance < 0.1 at the excitation wavelength were selected for further fluorescence spectroscopy studies to avoid the inner filter effect [25]. Steady-state fluorescence measurements were performed in a PTI QuantaMaster™ 8075-21 HORIBA spectrofluorometer (HORIBA Scientific, Japan), equipped with a 75-W short arc Xenon lamp (UXL-75XE, Ushio Inc), and a single emission/excitation monochromator. Fluorescence intensity was recorded, under stirring, in counts per second (cps), at 25 ± 0.1 °C, in 0.5 cm quartz cuvettes, 1 nm step-size and an integration time of 0.1 s. An average of three fluorescence emission spectra were collected for each sample. Data analyzed using ORIGIN pro 11.0 software (OriginLab Corporation, Northampton, MA, USA).

### 2.3. Peptide Synthesis

The peptides BP100 and W-BP100 were manually synthesized by solid-phase peptide synthesis (SPPS) using the standard 9-fluorenylmethoxycarbonyl (Fmoc)/tBu orthogonal protection scheme, on a Fmoc-Rink Amide MBHA resin (100–200 mesh, 0.52 mmol g^−1^, Novabiochem, Merck, Germany) [26]. Briefly, the resin was previously swelled in dimethylformamide (DMF, Sigma-Aldrich) and dichloromethane (DCM, Fischer Scientific, Hampton, NH, USA) for 30 min in each case. The Fmoc group was then removed from the resin using 20% piperidine (Sigma-Aldrich, HPLC grade) in DMF for 20 min, to release the free primary amine group required for subsequent coupling to the C-terminal of the N^α^-Fmoc-protected amino acid (Fmoc-AA-OH). Once successful removal of the Fmoc group was confirmed by the Kaiser, or ninhydrin, test [26], the coupling was promoted by addition, to the resin, of a solution containing 5 molar equivalents (eq) of the Fmoc-AA-OH (Bachem, Bubendorf, Switzerland), 5 eq of *N*,*N*,*N*′,*N*′-tetramethyl-*O*-(1*H*-benzotriazol-1-yl)uranium hexafluorophosphate (HBTU, NovaBiochem, Merck) and 10 eq of N-ethyl-N,N-diisopropylethylamine (DIEA, Sigma-Aldrich) in DMF, and allowing the reaction to proceed for 1 h at room temperature (r.t.) under stirring. After washing the resin 3–4 times with DMF, and also with DCM, the Kaiser test was done to confirm quantitative coupling of the Fmoc-AA-OH, after which the Fmoc group was removed with piperidine as before, to prepare the peptidyl-resin for the coupling of the next Fmoc-AA-OH. This coupling/deprotection cycle was repeated until the full amino acid sequence was built in the C→N direction, and the Kaiser test was done after all steps, which were repeated if the test did not return the expected result. Once the peptide sequence was completed, the peptides were detached from the resin using a cleavage cocktail containing 87.5% trifluoroacetic acid (TFA, Sigma-Aldrich), 2.5% triisopropylsilane (TIS, Sigma-Aldrich), 5% (*v*/*v*) phenol (Sigma-Aldrich) and 5% deionized water, for 2 h at r.t., under stirring. This solution was filtered and diluted in tert-butyl methyl ether (MTBE, Riedel-de Haën, Germany), and the peptide solutes were extracted from the organic layer with deionized water. The aqueous layer was freeze-dried and the crude peptides thus obtained were purified by reverse phase high performance liquid chromatography (RP-HPLC), on Hitachi-Merck LaPrep Sigma system (VWR, Radnor, PA, USA) equipped with an LP3104 UV detector and an LP1200 pump, employing a reverse-phase C18 column (250 × 25 mm ID and 5 µm pore size, Merck, Kenilworth, NJ, USA). The peptides were eluted using 0.05% aqueous TFA as solvent A and acetonitrile (ACN, Carlo Erba, Barcelona, Spain) as solvent B. A gradient elution of 10–35% (*v*/*v*) and 20–40% (*v*/*v*) of B in A was applied to isolate BP100 and W-BP100, respectively, for 60 min at 15 mL min^−1^ flow rate. The pure peptide fractions were pooled, freeze-dried and stored at −20 °C until further use. Final peptide purity degrees were determined by analytical HPLC using a Hitachi–Merck LaChrom Elite (VWR) system equipped with a quaternary pump, a thermostated automated sampler, and a diode array detector. Analyses were performed with a reverse-phase C18 column (150 × 4.6 mm ID and 5 mm pore size, Merck) at a 1 mL min^−1^ flow rate using a 1–100% gradient of B in A, for 30 min, with detection at 220 nm. Peptide structure was confirmed by electrospray ionization-ion trap mass spectrometry (ESI-IT MS, Thermo Finnigan, CA, USA).

### 2.4. Antibacterial Activity

The minimum inhibitory concentration (MIC) and minimum bactericidal concentration (MBC) values for W-BP100 and BP100 against susceptible reference strains (*Pseudomonas aeruginosa* ATCC 27853, *Escherichia coli* ATCC 25922, *Staphylococcus aureus* ATCC 29213, and *Enterococcus faecalis* ATCC 29212) were determined by the broth microdilution method, following the Clinical and Laboratory Standards Institute (CLSI) guidelines [27]. Briefly, two-fold serial dilutions from a peptide stock solution (in water) were prepared in 96-well round-bottom plates, in cation-adjusted Mueller–Hinton Broth (MHB2, Sigma-Aldrich), and ranging from 0–433 µmol dm^−3^ for BP100 and 0–383 µmol dm^−3^ for W-BP100 (0–614 µg mL^−1^ for both peptides). Each bacterial suspension was then prepared in MHB2 and inoculated in order to achieve a final concentration of approximately 5 × 10^5^ colony forming units (CFUs) mL^−1^ per well. The microplates were incubated at 37 °C for 24 h. The MIC was determined as the lowest peptide concentration at which no visible bacterial growth was detected by naked eye. The MBC was the lowest concentration at which no bacterial growth occurred after plating 10 µL of bacterial suspension from the wells at and above the MIC on Mueller–Hinton Agar (MHA), and incubating at 37 °C for 24 h [28]. At least two independent experiments were performed.

### 2.5. Preparation of Large Unilamellar Vesicles

LUV composed of POPC:POPG (1:1) and POPC were prepared as membrane models of bacteria and mammalian cells, respectively. Lipid films were prepared as described elsewhere [29], by dissolving the lipids in chloroform, followed by solvent evaporation under a nitrogen stream. The lipid films were kept under vacuum for 3 h to remove any trace of solvent and the resulting lipid films were resuspended in HEPES buffer. The lipid suspension was further vortexed to obtain multilamellar large vesicles (MLV). The MLV were subjected to five freeze-thaw cycles and equilibrated at r.t. for 30 min. The lipid suspension was extruded 10 times using an extruder from LIPEX Biomembranes (Vancouver, Canada), attached to a CLIFTON thermostatic circulating water bath, at r.t. (above the phase transition temperature of POPC and POPG, ~−2 °C), through polycarbonate filters of 0.1 µm pore size (Whatman, GE Healthcare, Maidstone, UK) to obtain the LUV. Size distribution of LUV was characterized by DLS at 37 ± 0.1 °C (Zetasizer nano ZS, Malvern Panalytical Malvern, UK), using a He-Ne laser at a wavelength of 633 nm, and a fixed scattering angle of 173°. The average particle size diameter of LUV suspensions was ~100 nm, with a polydispersity index (PdI) < 0.1. Phospholipid concentration was determined by phosphate quantification adapted from the method of Bartlett et al. [30].

To prepare CF-loaded LUV for leakage assays, 20 mmol dm^−3^ lipid films were resuspended in 0.5 mL of 50 mmol dm^−3^ CF, previously solubilized in HEPES buffer and pH adjusted to 7.4. The free CF was removed by gel filtration, eluting with HEPES buffer through a Sephadex^®^ G-50 Fine column (Sigma-Aldrich) [15]. The LUV were collected at the column void volume and used within 1 week, with CF-loaded LUV showing residual leakage in the absence of peptides.

### 2.6. Photophysical Properties of Peptides in Aqueous Solution

The peptides were solubilized in HEPES buffer and their concentration was determined using the Thermo Scientific^TM^ NanoDrop^TM^ One Microvolume UV–vis spectrophotometer (Thermo Fisher Scientific, Waltham, MA, EUA), based on the absorbance of peptide’s backbone at 205 nm. Peptide concentration was calculated using the Scopes method from the NanoDrop One Protein A205 application, using the absorbance ratio A280/A205, to account for Tyr and Trp side chain absorbance. The linear dependence of absorption and fluorescence intensity over a peptide concentration range of 0–90 µmol dm^−3^ was studied as described by Ferre et al. [13]. The fluorescence properties of BP100 were evaluated at an excitation wavelength of 275 nm, in the wavelength range from 285–400 nm, using excitation/emission slit widths of 2.5 nm, while W-BP100 was excited at 280 nm, in the wavelength range from 300–450 nm, using excitation/emission slit widths of 2.0 nm. 

### 2.7. Partition Constants

The interaction of BP100 and W-BP100 with lipid membrane models was evaluated using steady-state fluorescence spectroscopy. We took advantage of the intrinsic fluorescence properties of the aromatic amino acids, Trp and Tyr, and their sensitivity to hydrophilic-lipophilic environments, to determine the partition of peptides into POPC:POPG (1:1) and POPC LUV [31]. Data were acquired at an excitation wavelength of 275 nm and wavelength range of 285–400 nm for BP100, and an excitation wavelength of 280 nm, in the range of 290–450 nm for W-BP100. Excitation/emission slits for BP100 with POPC:POPG (1:1) of 3/4 nm and POPC of 4/5 nm, while for W-BP100 with POPC:POPG (1:1) of 2.5/2.5 and POPC of 3/3 nm. The partitioning of peptides into the lipid membranes was assessed by titrating 9 µmol dm^−3^ of each with 0–1.00 mmol dm^−3^ LUV, in HEPES buffer pH = 7.4, at 25 ± 0.1 °C. Fluorescence intensity was recorded after 10 min of incubation upon each lipid addition. Fluorescence intensity was corrected for dilution and, in the case of BP100, for scattering light due to the low quantum yield of Tyr when compared to Trp, and subsequently reduced fluorescence emission intensity of BP100. 

### 2.8. Membrane Saturation Studies

W-BP100 was evaluated for its ability to induce membrane saturation of POPC:POPG (1:1) LUV using steady-state florescence spectroscopy. Fluorescence intensity was acquired at an excitation wavelength of 290 nm, in the wavelength range of 300–450 nm and excitation/emission slits of 4/5 nm. Several lipid concentrations (0, 0.015, 0.030, 0.060, and 0.100 mmol dm^−3^) were titrated with 0–9 µmol dm^−3^ W-BP100 in the presence of 100 mmol dm^−3^ acrylamide (Sigma-Aldrich), as described elsewhere [13,32]. To avoid acrylamide dilution, the lipid suspensions were prepared with the same acrylamide concentration, and samples were incubated 10 min before recording the fluorescence emission spectra. 

### 2.9. Fluorescence Acrylamide Quenching

Acrylamide was used to study the fluorescence quenching of peptides in aqueous solution (HEPES buffer) or in the presence of 1 mmol dm^−3^ POPC:POPG (1:1) and POPC LUV. 9 µmol dm^−3^ of each peptide in aqueous solution or mixed with LUV were titrated with increasing concentrations of acrylamide up to 250 mmol dm^−3^. For BP100, the fluorescence intensity was recorded with an excitation wavelength of 275 nm, wavelength range of 285-400 nm and excitation/emission slits of 6/7 nm (aqueous solution) and 4/5 nm (LUV). For W-BP100, the fluorescence intensity was recorded at an excitation wavelength of 290 nm, to reduce the quencher/fluorophore light absorption, a wavelength range of 300–450 nm and excitation/emission slits of 4/4 nm (aqueous solution) and 3/3 nm (LUV). Fluorescence intensity of BP100 was corrected for the light absorption of acrylamide at the peptide’s excitation wavelength [33].

### 2.10. Aggregation of LUV Using DLS

The effect of BP100 and W-BP100 on LUV aggregation was evaluated by DLS, as described elsewhere [13]. Peptide concentrations ranging from of 0–15 µmol dm^−3^ were incubated for 10 min in the presence of 100 µmol dm^−3^ POPC:POPG (1:1) or POPC LUV. The particle-size diameter and the polydispersion index were measured in 1 cm length plastic cuvettes, at 25 ± 0.1 °C, upon an equilibrating time of 60 s.

### 2.11. Membrane Permeabilization

Permeabilization of LUV by BP100 and W-BP100 was carried out using CF-loaded LUV in HEPES buffer, prepared as described in Section 2.6 [15]. Briefly, 60 µmol dm^−3^ CF-loaded POPC:POPG (1:1) were incubated with 0–9.4 µmol dm^−3^ of each peptide, at 25 ± 0.1 °C. CF release was monitored at an excitation wavelength of 492 nm and emission wavelength of 517 nm, using an excitation/emission slits of 1/3 nm for both peptides. Total CF release was obtained by using 0.1% (*v*/*v*) Triton^TM^ X-100 (Sigma-Aldrich). For leakage kinetics, 5 points per second were acquired along 30 min. Experimental data were corrected for dilution. Concentration–response curves were obtained by plotting the percentage of CF leakage upon 30 min incubation as function of peptide concentration. Membrane permeabilization efficiency was determined by the peptide concentration that induces 50% leakage of CF from the model membranes (LC_50_).

## 3. Results and Discussion

### 3.1. Peptide Synthesis and Photophysical Characterization

Peptides were successfully obtained with HPLC purity > 95%, as given in the Supplementary Material (Table S1; Figures S1–S4). The maximum absorption and excitation wavelength of W-BP100 at 25 °C was 280 nm (Figure S5C), while the maximum fluorescence emission intensity was observed at 353 nm (Figure S6B), which agrees with the photophysical properties reported for free Trp [34,35]. The molar absorptivity coefficient obtained, 7370 mol dm^−1^ cm^−1^, was similar to the sum of the molar absorptivity coefficients of free Tyr and Trp at 280 nm and 25 °C (Figure S5D) [34]. The absorbance of W-BP100 in aqueous solution correlated linearly with peptide concentration up to 90 µmol dm^−3^ (Figure S5D), suggesting that W-BP100 do not aggregate in aqueous solution within this concentration range. The parent peptide BP100 exhibited the same absorbance and fluorescence properties as reported in the literature [13] (Figures S5A,B, and S6A).

**Figure 1:**
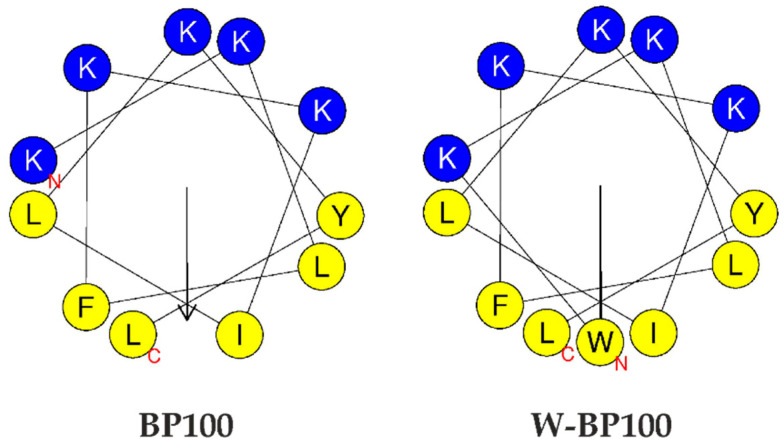
Helical wheel projections of BP100 and W-BP100. Polar amino acid residues are in blue and non-polar ones in yellow. C and N represent the amino and carboxyl termini, respectively, and the arrows indicate the directions and magnitudes of the hydrophobic moments. Amino acid residues are represented by the single letter code as defined by the IUPAC-IUBMB guidelines on nomenclature and symbolism for amino acids and peptides. Helical wheel projections were generated in the HELIQUEST software [38].

BP100 is a positively charged (overall charge of +6, considering the charge of Lys residues and the amine group of the N-terminus at neutral pH) and amphipathic peptide, able to acquire an α-helical conformation in model membranes, as confirmed early by circular dichroism and molecular dynamics studies [15,36]. The helical wheel projection of W-BP100 was generated in the HELIQUEST program to evaluate its amphipathic properties [37]. The addition of a single Trp at the N-terminal position of the peptide should not substantially affect the amphipathic nature of the parent peptide, given the presence of well-defined hydrophobic and hydrophilic faces (Figure 1). This N-terminal Trp, known to have high affinity for the membrane–water interface of lipid membranes [22], is positioned in the hydrophobic domain.

### 3.2. Antibacterial Activity of Peptides

The antibacterial activity of W-BP100 and its parent peptide BP100 was assessed by determination of the respective MIC and MBC values against susceptible Gram-positive and Gram-negative bacterial species. W-BP100 showed a substantial improvement in the antibacterial activity against Gram-positive species (1.5–3 µmol dm^−3^), as compared to the parent peptide (27–216 µmol dm^−3^), while similar antibacterial activity against Gram-negative bacteria was observed for both peptides (Table 1). The minimum inhibitory concentration (MIC) and maximum inhibitory concentration (MBC) displayed by the parent peptide BP100 are within the range previously reported for phytopathogenic and human pathogenic Gram-negative bacteria [14,15]. Importantly, W-BP100 was bactericidal at the MIC (Table 1).

Similar to W-BP100, Torcato et al. observed that RW-BP100 was substantially more active than BP100 against Gram-positive species, in the same range of MIC values as we obtained for W-BP100 [15]. RW-BP100 was rationally designed to improve the bioactivity of BP100 and, apart from the Tyr substitution by Trp near the peptide’s C-terminus, it holds an additional substitution of native Lys by Arg. This conserves the overall positive charge, allowing the establishment of H-bonds with the polar headgroups of phospholipids, while enhanced cation–π interactions contribute to the stronger antibacterial activity of this peptide [15]. With our results it is possible to state that a similar enhancement of the antibacterial activity is achieved by the incorporation of a single Trp to the N-terminus of BP100, instead of multiple amino acid substitutions [8,9]. Our results show that the combination of Trp and Arg residues are not exclusively required to improve the bioactivity of the parent peptide BP100.

At a molecular level, W-BP100, holding the same cationic charge as BP100 and RW-BP100, promotes electrostatic interactions with the negatively charged components of the bacterial cell envelope, e.g., lipopolysaccharides in Gram-negative bacteria, lipoteichoic acids in Gram-positive bacteria and the phospholipid headgroups of PG phospholipids. Furthermore, W-BP100 contains the particular features of Lys and Trp residues, conferring the ability to establish cation–π interactions between basic residues (e.g., Lys and Arg) and aromatic residues (e.g., Trp, Phe, and Tyr). These interactions with aromatic residues are important for peptide self-association and deeper insertion within membranes by shielding the charge of cationic residues [39,40].

### 3.3. Peptide–Membrane Interactions with LUV

**Figure 2:**
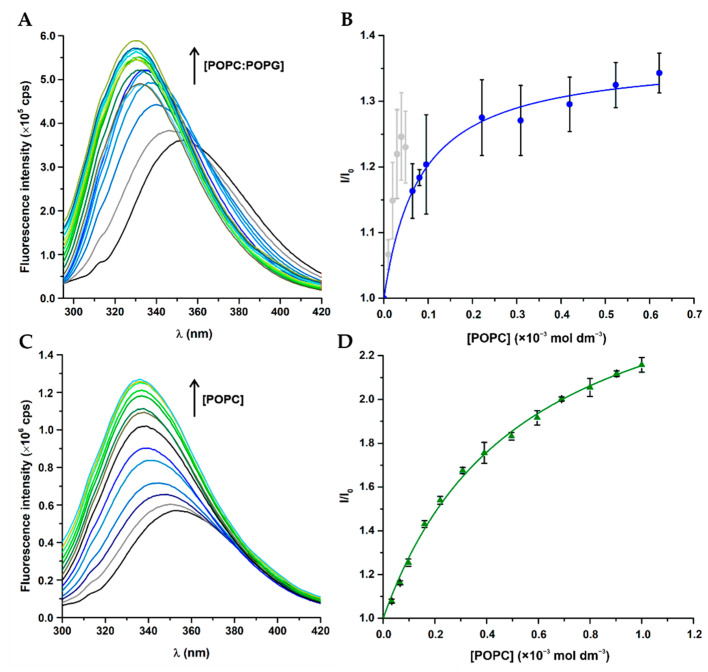
Membrane partitioning of W-BP100 in anionic and zwitterionic model membranes determined by steady-state fluorescence spectroscopy. Representative fluorescence emission spectra of W-BP100 in presence of (**A**) POPC:POPG (1:1) and (**C**) POPC LUV. (**B**,**D**) Spectral lines of the non-linear fitting of experimental data to the simple partition model in Equation (2). Data points and error bars are the mean ± SD of three independent experiments.

The partition of peptides into lipid membranes can be determined by the fluorescence properties of aromatic residues such as Trp and Tyr. Besides working as an intrinsic fluorescent probe to study peptide-membrane interactions, Trp is quite sensitive to the polarity of the surrounding environment, eliciting a well-known preference for the membrane–water interface [41]. W-BP100 partition into membrane models was determined by following the steady-state fluorescence properties of Trp in the absence and presence of anionic POPC:POPG (1:1) and zwitterionic POPC LUV, as models of bacterial and eukaryotic cell membranes, respectively (Figure 2A,C). In both membrane models, W-BP100 exhibited an increase of the fluorescence emission intensity, concomitant with a hypsochromic shift of fluorescence emission maximum upon titration with increasing concentrations of LUV. It varied from λ_em_ = 353 nm in the absence of lipids to λ_em_ = 331 nm and 336 nm in 1 mmol dm^−3^ POPC:POPG (1:1) and POPC, respectively.

The partition constant, *K*_p_, of the peptides was determined by the simple partition model, as described by Santos et al. [31] and Ferre et al. [13]

(1) $$\frac{I}{I_{W}}=\frac{1+K_{p}\gamma_{L}\frac{I_{L}}{I_{W}}\left[L\right]}{1+K_{L}\gamma_{L}\left[L\right]}$$

by fitting Equation (1) to the experimental data, where $I_{L}$ and $I_{W}$ are the fluorescence intensities of the peptides if all peptide is in the lipid or aqueous phase, respectively. $\gamma_{L}$ is the phospholipid molar volume (0.763 mol^−1^ dm^3^) and [L] is the lipid concentration. 

As emission spectral shifts can be a consequence of peptide–lipid interactions, the integrated spectral intensity is commonly preferred over single wavelength intensity [41,42]. In this work, full spectra were always used although the results were similar to those obtained by using a single emission wavelength (results not shown).

For W-BP100 the expected hyperbolic-like curve was obtained in POPC LUV (Figure 2D), while in POPC:POPG (1:1) upward deviations to the simple partition model were obtained at high peptide/lipid (P/L) ratios, which were excluded from the fitting of Equation (1) (Figure 2B, light grey data points).

These deviations were previously described as membrane saturation events for other CAMP, such as omiganan (ILRWPWWPWRRK-NH_2_), an indolicidin-related peptide [32]. The partition of W-BP100 into the anionic POPC:POPG (1:1) model, *K_p_* of (16.0 ± 1.8) × 10^3^, was greatly enhanced when compared to the zwitterionic POPC model, *K*_p_ of (2.4 ± 0.2) × 10^3^ (Table 2).

**Figure 3:**
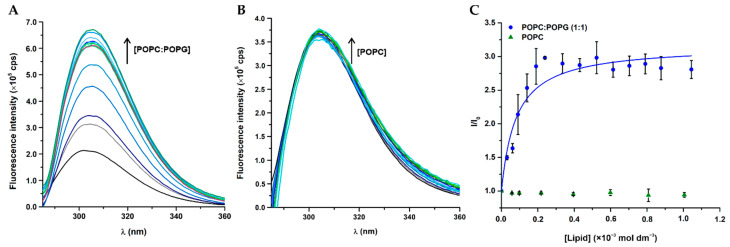
Membrane partitioning of BP100 in anionic and zwitterionic model membranes determined by steady-state fluorescence spectroscopy. Representative fluorescence emission spectra of BP100 as a function of (**A**) POPC:POPG (1:1) and (**B**) POPC LUV. (**C**) Spectral lines of the non-linear fitting of experimental data to the simple partition model in Equation (1). Data points and error bars are the mean ± SD of two to three independent experiments.

The partition of BP100 into anionic LUV (Figure 3) followed the trend reported in the literature [13,15]. Nevertheless, the fitting of Equation (1) for BP100 fluorescence data in the presence of the zwitterionic model was not possible, because no significant spectral changes were detected.

Both peptides have a similar *K*_p_ into POPC:POPG (1:1) LUV, despite their distinct partition profiles, suggesting similar affinity for negatively charged membranes (Table 2). Nonetheless, accurate determination of the partition constant of W-BP100 in anionic LUV was difficult although it was possible to conclude that this partition was greatly enhanced relatively to the neutral system.

**Figure 4:**
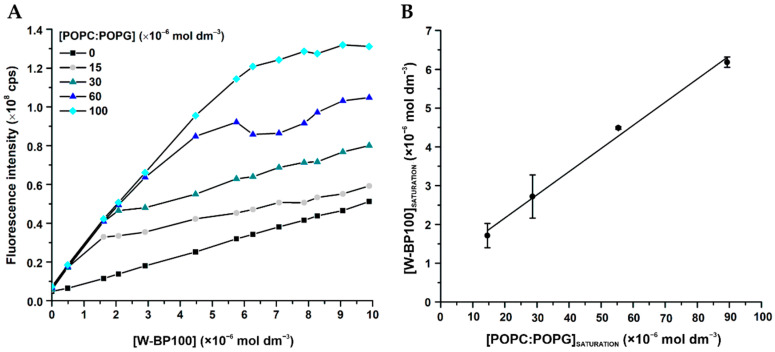
W-BP100-induced membrane saturation in POPC:POPG (1:1) LUV. (**A**) Titration of POPC:POPG (1:1) LUV in HEPES buffer, containing 100 mmol dm^−3^ acrylamide, with increasing concentrations of W-BP100, at 25 ± 0.1 °C, as described by Melo et al. [32]. The breaking points in each curve, where a change of slope is observed, represent membrane saturation points. The fluorescence spectra of each titration are reported in the supplementary material (Figure S7). (**B**) Plotting of W-BP100 and POPC:POPG (1:1) concentrations at saturation points, and fitting to the membrane saturation model Equation (2). Data points and error bars represent the mean ± SD of three independent experiments.

To gain information on the membrane saturation induced by W-BP100, saturation events were assessed in anionic LUV in a similar way as described by Melo and co-workers [32]. We evaluated the occurrence of membrane saturation by titrating different lipid concentrations with increasing peptide concentrations in the presence of acrylamide (aqueous phase quencher) to better distinguish the differences between fluorescence intensity of the peptide in the absence and presence of POPC:POPG (1:1) LUV. For each lipid concentration tested, the fluorescence intensity of W-BP100 presented two regions with distinct slopes (Figure 4A): a higher slope at high P/L ratios, consistent with strong peptide membrane interactions, and a second slope at low P/L ratios, corresponding mostly to free peptide present in the aqueous phase, available to be efficiently quenched by acrylamide. The breaking points in each curve correspond to the peptide and lipid concentrations at which membrane saturation happens, thus when no more lipid is available to accommodate increasing concentrations of peptide. By applying the membrane saturation model equation, Equation (2), proposed by Melo et al. [32], the $K_{p}$ of W-BP100 at membrane saturation conditions was calculated as

(2) $$\left[P\right]=\frac{\sigma }{K_{p}\gamma_{L}}+\sigma \left[L\right]$$

where [P] and [L] are the peptide and lipid concentrations, respectively, at membrane saturation points and σ is the P/L proportion in a saturated membrane. While the simple partition model view the peptide as a volumeless entity, therefore unable to induce the saturation of lipid membranes [31], the membrane saturation model assumes that the amount of peptide in the lipidic phase is directly proportional to its volume, and therefore to the amount of available lipid in solution [32].

Our results show a linear correlation between peptide and lipid concentrations at membrane saturation (Figure 4B), validating the occurrence of membrane saturation events in anionic membranes in the presence of W-BP100. Correlation between membrane saturation and the partition curve was further corroborated, as saturation pairs from partition curves (results not shown) were in agreement with the saturation pairs from *I* vs. [P] curves (Figure 4A).

The obtained *K*_p_ was very high, but the obtained P/L ratio (σ) at membrane saturation conditions, σ = 0.06, seem plausible and robust. Considering that *K*_p_ is calculated from the reciprocal of a relatively small intercepted, which makes this an unsuitable method for determination of very large partition constants, it is expected that this value has large associated errors [43].

Melo and co-authors described a strong affinity to anionic membranes while inducing their saturation for the cationic and Trp-rich peptide omiganan (overall charge + 5) in anionic POPC:POPG (1:2) and POPC:POPG (1:4) LUV [32]. These features were also reported for others CAMP such as indolicidin [44], cecropin P1 [45], melittin [46], and inclusively BP100 in anionic POPC:POPG (1:2) LUV [13,47]. We show that W-BP100 behaved similarly, eliciting a strong affinity to anionic LUV while inducing membrane saturation, as validated by the membrane saturation model [32].

The deviations to the simple partition model, observed for W-BP100, occurred with concomitant high increase of fluorescence intensity at low lipid concentrations, suggesting strong interactions with anionic membranes. This is driven by the high positive charge of W-BP100 (overall charge + 6) which correlates with its high affinity to anionic LUV as consequence of strong electrostatic interactions. Additionally, an enhancement and blue shift of fluorescence emission maximum of Trp was observed in both membrane models (Figure 2A,C). This is a common property of Trp residues located in hydrophobic environments [41], such as those positioned in protein transmembrane domains [23].

W-BP100 showed high antibacterial activity and strong partition into anionic membranes, and considering that it only differs from BP100 in one single additional hydrophobic amino acid, the presence of a Trp at the N-terminus seems to greatly impact peptide-membrane interactions, which is relevant for the rational design of more potent analogues of known CAMP. Overall, our data were consistent with data reported for BP100 [13], and besides showing the ability of W-BP100 to induce membrane saturation in anionic LUV, also validates its high affinity and strong partition towards negatively charged membranes.

### 3.4. Membrane Location of Peptides in LUV

Quenching studies, using the hydrophilic quencher acrylamide, provide information on the peptide exposure to the aqueous environment, given the presence of fluorescent amino acids in the peptide’s sequence, such as Trp and Tyr [41]. When fluorophores are accessible to an aqueous solvent, acrylamide quenches its fluorescence allowing to estimate its preferential location, based on the calculated Stern–Volmer constant or collisional constant, *K*_SV._ The quenching of peptides in the absence and presence of lipids was fitted according to the Stern–Volmer equation for collisional quenching, Equation (3) [41]

(3) $$\frac{I_{0}}{I}=1+\;K_{\mathrm{SV}}\;[Q]$$

where *I*_0_ and *I* are the fluorescence intensities of peptides measured in the absence and presence of acrylamide ([Q]) respectively, and $K_{\mathrm{SV}}$ is the Stern–Volmer constant or collisional constant.

**Figure 5:**
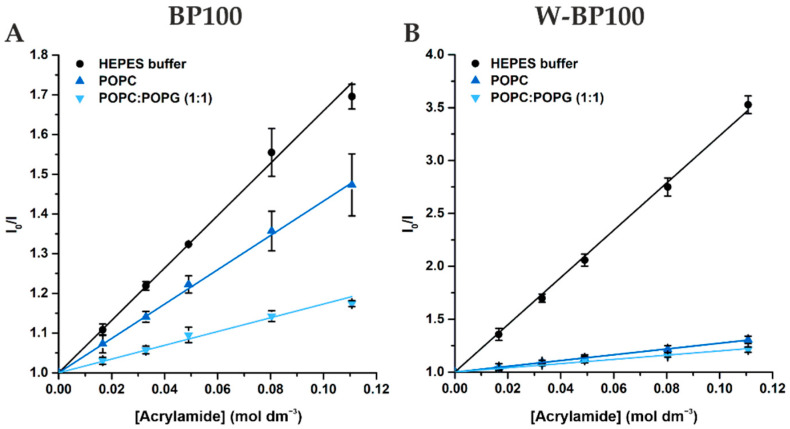
Acrylamide quenching of peptide fluorescence in aqueous solution and in the presence of LUV. Quenching of fluorescence of (**A**) BP100 and (**B**) W-BP100 in HEPES buffer and in the presence of POPC:POPG (1:1) and POPC LUV. Experimental data was fitted to the Stern–Volmer equation for collisional quenching, Equation (3). Data points and error bars are the Mean ± SD of three independent experiments. The fluorescence spectra for each condition are reported in the supplementary material (Figure S8).

The results obtained for both peptides followed a linear Stern–Volmer correlation up to 111 mol^−1^ dm^3^ of acrylamide with a correlation coefficient R^2^ always equal or higher than 0.9997 (Figure 5).

W-BP100 was more efficiently quenched by acrylamide in the absence of model membranes, similar to the parent peptide (Figure 5), and as demonstrated by the higher Stern-Volmer constant values obtained (Table 3). These results suggest that their aromatic residues are less accessible to the aqueous environment, being inserted within the lipid bilayer. Additionally, the fluorescence of both peptides is less efficiently quenched in the presence of POPC:POPG (1:1) comparing to POPC LUV suggesting a better insertion into negatively-charged membranes.

Nevertheless, the quenching efficiency of BP100 in aqueous and lipidic phases was less pronounced than that observed for W-BP100 (Table 3). The results for the BP100 are consistent with data reported in the literature [15]. For W-BP100, the acrylamide quenching efficiency in presence of POPC:POPG (1:1) and POPC LUV was substantially lower than in aqueous solution (Table 3). These results show that in the presence of both membrane models, the Trp residue of W-BP100 is mostly inaccessible to the aqueous phase, clearly preferring hydrophobic environments.

In anionic membranes, BP100 is known to adopt a mobile and membrane parallel α-helical conformation, occupying a surface state position at the membrane–water interface [48,49]. While the hydrophilic domain of the α-helix interacts with the anionic headgroups of phospholipids, the hydrophobic domain interacts with the acyl chains, promoting peptide insertion into the lipid bilayers [36,50]. A similar behavior was observed for RW-BP100 in anionic LUV by Torcato et al. [15]. Given the similarities of W-BP100 with these peptides and knowing that most small amphipathic peptides with Trp residues adopt a membrane-bound α-helix secondary structure [15,51,52,53,54], it is likely that W-BP100 will adopt this conformation in anionic membranes. Additionally, the presence of a hydrophobic Trp residue at its N-terminus, located in the hydrophobic domain of the predicted amphipathic conformation (Figure 1), facilitates the insertion of W-BP100 into lipid membranes, as validated by the acrylamide quenching studies (Figure 5). 

### 3.5. Aggregation and Membrane Permeabilization Studies

BP100 is known to induce aggregation of anionic LUV, which is closely associated to its cationic nature and the ability to neutralize the membrane surface charge [13]. As W-BP100 and BP100 have an equal number of positively charged residues, we evaluated its ability to induce the aggregation of zwitterionic and anionic LUV using DLS. No effect on the size of POPC LUV was observed in the presence of any of the peptides (Figures S10 and S11). 

**Figure 6:**
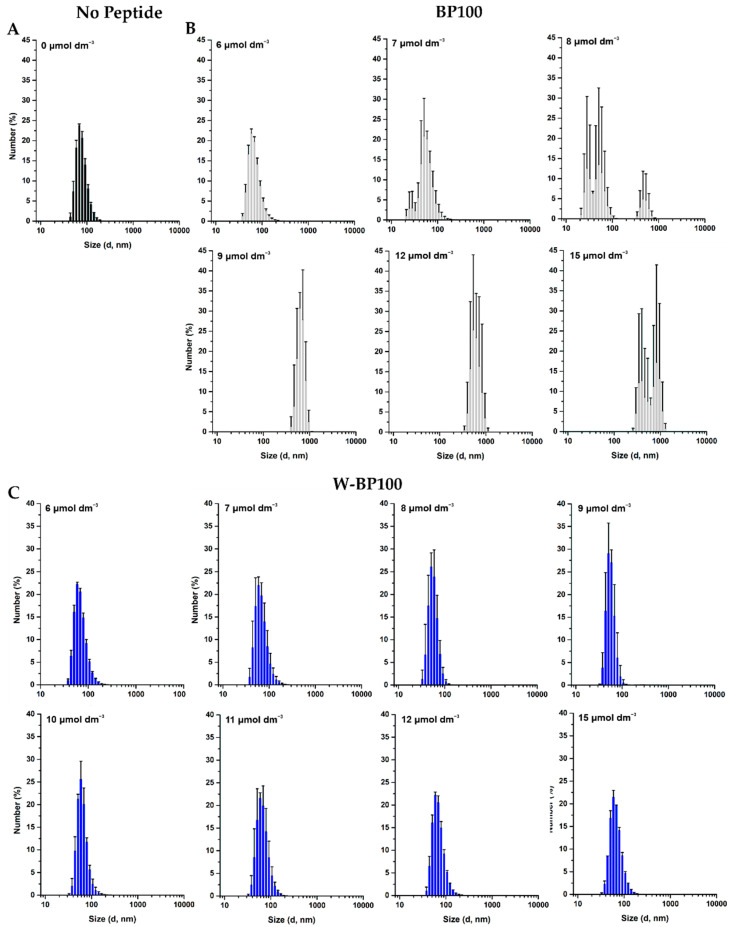
Size distribution of anionic LUV in the presence of increasing peptide concentrations. Number-weighted size distribution of 100 µmol dm^−3^ POPC:POPG (1:1) LUV in (**A**) absence and presence of 0–15 µmol dm^−3^ (**B**) BP100 and (**C**) W-BP100. Data are the mean of size distribution ± SD of at least three independent experiments.

BP100 induced aggregation of POPC:POPG (1:1) LUV at and above 8 µmol dm^−3^ (P/L of 0.08) (Figure 6B), consistent with data reported in the literature [13]. This aggregation is promoted by electroneutrality favoring vesicle aggregation by canceling the electrostatic repulsion between the anionic vesicles [13].

Unexpectedly, analyzing the number-weighted size distribution for W-BP100 in anionic LUV, no aggregation was observed at membrane saturation conditions (6 µmol dm^−3^ and P/L of 0.06) (Figure 6C). 

Instead, a reduction of the vesicle size was observed at concentrations close to membrane saturation (7–11 µmol dm^−3^), with a size distribution smaller than 100 nm. In this concentration range, some aggregation was observed, as demonstrated by analyzing the intensity-weighted size distribution, although the number of particles with higher dimensions was negligible (Figure S9C), overall supporting a detergent-like action for this peptide. At higher peptide concentrations a size distribution around 100 nm was again observed (Figure 6C).

We propose that W-BP100 follows a two-step process interaction behavior initiated with electrostatic attraction and adsorption at the membrane surface and charge reduction, possibly followed by peptide translocation across the lipid bilayers, resulting in a sequential aggregation and disaggregation of POPC:POPG (1:1) LUV, as proposed by Bagheri et al. for the Trp-rich peptide HHCs [55]. Overall, this hypothesis would explain why W-BP100 does not induce vesicle aggregation at higher P/L ratios (Figure 6C), as observed for the parent peptide (Figure 6B). However, further experiments are required to elucidate the MOA of W-BP100, namely, to evaluate its effect on the surface charge of LUV, peptide self-association and/or translocation within membranes, and if pore formation occurs. 

Considering that W-BP100 showed a strong affinity towards anionic membranes, membrane permeabilization was determined for both peptides, using CF-loaded POPC:POPG (1:1) LUV. The percentage of CF leakage was calculated according to Equation (4)

(4) $$\%\;CF\;Leakage=\;\frac{I_{t}-I_{0}}{I_{\max}-I_{0}}\;\times 100$$

where $I_{t}$ is the fluorescence intensity in the presence of each peptide at time *t*, $I_{0}$ and $I_{\max}$ correspond to the fluorescence intensity in the absence of peptide (negative control) and 100% release of CF upon addition of Triton X-100 (positive control), respectively.

Concentration–response curves were obtained by plotting the percentage of CF leakage upon 30 min incubation as function of peptide concentration. Data were fitted to the following non-linear sigmoidal logistic equation

(5) $$y=Bottom+\;\frac{Top-Bottom}{1+{\left(x/{\mathrm{LC}}_{50}\right)}^{p}}$$

where y corresponds to CF leakage at a given peptide concentration x, LC_50_ corresponds to the peptide concentration that induces 50% CF release (leakage efficiency), *Top* and *Bottom* refers to the upper and lower boundaries (constrained to values < 100% and >0%, respectively), and *p* is the hill coefficient [56].

**Figure 7:**
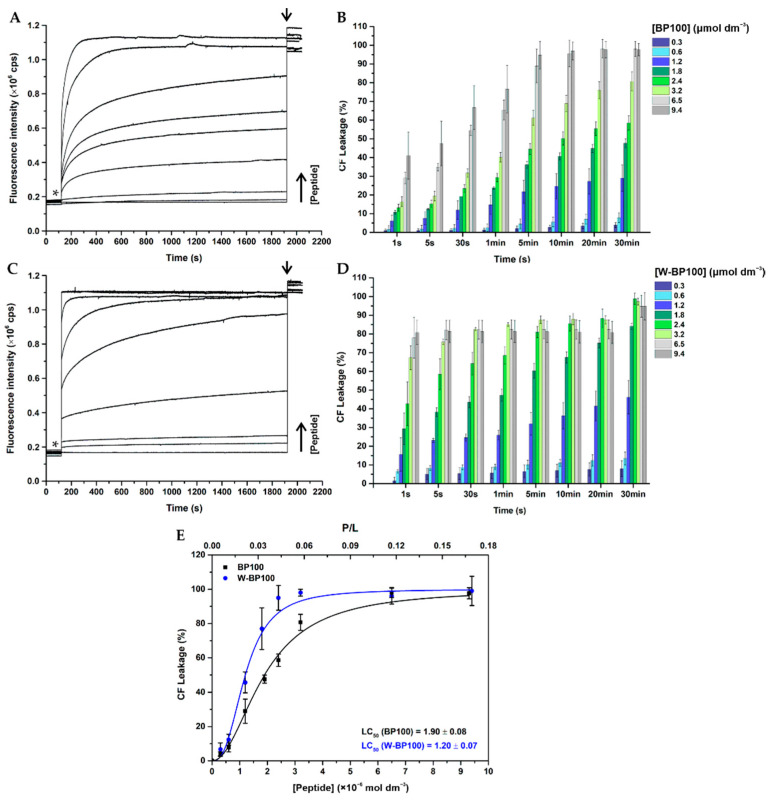
Peptide-induced permeabilization of anionic model membranes. (**A**,**B**) Leakage kinetics of carboxyfluorescein (CF) release from 60 µmol dm^−3^ CF-loaded POPC:POPG (1:1) LUV in the presence of increasing concentrations of (**A**,**B**) BP100 and (**C**,**D**) W-BP100, along 30 minutes of incubation in HEPES buffer, at 25 ± 0.1 °C. Asterisks and arrows mark the time-points of peptide and Triton-X addition, respectively. (**E**) Percentage of CF release as function of peptide concentration and P/L ratio. LC_50_ represents the peptide concentration that promotes 50% leakage of CF. Data points and error bars represent the mean ± SD of at least two independent experiments.

W-BP100 induced the leakage of CF from anionic POPC:POPG (1:1) LUV in a concentration-dependent manner, similarly to BP100 (Figure 7) [13,15,16,57]. The peptide concentration that promotes 50% release of CF (LC_50_) was slightly lower for W-BP100 (1.20 ± 0.07 µmol dm^−3^ and P/L of 0.02), than for the parent peptide (1.90 ± 0.08 µmol dm^−3^ and P/L of 0.03), correlating with the more potent antibacterial activity of the former (Figure 7E). 

The leakage kinetics of W-BP100 displayed two distinct profiles dependent on the P/L ratio, as also reported for BP100 [13,16,57]. W-BP100 produced a reduced and gradual CF release at P/L ratios < 0.02, and at concentrations close to the lower MIC values (0.75 µmol dm^−3^), while increased and fast membrane leakage occurred at higher P/L ratios, at concentrations around the higher MIC values (3 µmol dm^−3^) (Figure 7D).

In addition, the leakage kinetics of W-BP100 presented a burst release of CF immediately upon peptide addition (Figure 7C), in contrast to BP100 (Figure 7A).The release of 100% of CF from anionic LUV occurred at P/L ≥ 0.04, close to the P/L ratio at which membrane saturation occurs (σ ~0.06) (Table 2), which is consistent with the MOA described for other CAMP, when a concentration threshold is reached at the membrane surface [47,58]. The membrane permeabilizing activity of W-BP100 follows the behavior of the analogue RW-BP100 reported by Torcato et al., inducing CF leakage from anionic vesicles at a higher rate than BP100 [15]. The authors also observed higher permeabilization of zwitterionic vesicles and an increase of the hemolytic activity comparing with the parent peptide. Preliminary data on the hemolytic activity of W-BP100 suggests a higher cytotoxicity in comparison to BP100 (data not published).

The high cationic nature of BP100 (overall charge + 6 at pH 7.4) is known to promote strong electrostatic interactions with anionic membranes, leading to membrane saturation, surface charge neutralization, membrane destabilization, vesicle aggregation, and permeabilization of model membranes and *Escherichia coli* in a concentration-dependent manner [13,14,36,57]. Its mechanism of action depends on the P/L ratio and membrane composition, namely on the PG content of anionic model membranes [13,57]. BP100 is known to trigger two distinct membrane leakage profiles, as early demonstrated by Ferre et al. [13] and later by Manzini et al. [57]. At low P/L ratios and low PG, BP100 induces lower and slow membrane permeabilization, coherent with a membrane thinning mechanism, while at high P/L ratios or high PG, close to membrane saturation conditions, it induces membrane disruption leading to high and instantaneous leakage of vesicles content, resembling a carpet-like mechanism [57].

Although the membrane permeabilization activity of W-BP100 also depends on the P/L ratio, its strong and faster permeabilization effect cannot be solely explained by the contribution of electrostatic interactions and its amphipathic character, since both peptides share an equal number of cationic residues and similar amphipathicity (Figure 1).Our data suggest that the presence of Trp at the N-terminus, in the hydrophobic domain of W-BP100, is significantly contributing to its marked ability for membrane insertion (Figure 5), membranolytic activity (Figure 7) and broader antibacterial activity (Table 1). Remarkably, and in contrast with the parent peptide, W-BP100 does not induce significant aggregation of anionic LUV at and above the P/L ratio of membrane saturation (P/L of 0.06) (Figure 6), despite having a strong membrane-permeabilizing effect. Given these results and the similarities of our peptide to the biophysical characterization of RW-BP100, we cannot exclude some toxicity associated with W-BP100, however, further experiments are required to evaluate the contribution of Trp on the cytotoxicity activity of this new peptide.

## 4. Conclusions

In the last decades, the lack of alternatives to clinically available antibiotics led to an urgent race towards the discovery of new and effective antimicrobial agents capable of surpassing bacterial resistance. CAMP are being widely explored to replace or complement current antibiotic therapies since they have been reported as promising antibacterial molecules. The study of the interactions of CAMP with lipid membrane models are, therefore, an essential piece of the intricate puzzle for the election of bioactive peptides as promising lead compounds to fight bacterial resistance.

The new cecropin A-melittin hybrid herein presented, W-BP100, is a strongly active CAMP against both Gram-negative and Gram-positive bacteria, and substantially more potent than the parent peptide BP100, in the latter case. Taken together, our data suggest that, despite both peptides have similar patterns of interaction with anionic vs. zwitterionic lipid vesicles, W-BP100 demonstrated a preferential insertion into lipid membranes, with a sequential aggregation and disaggregation of liposomes, according to the P/L ratio, inducing a strong and fast membrane permeabilization, in clear contrast with the parent peptide. 

In summary, we highlight that the simple addition of a Trp residue to the N-terminus of a CAMP, such as BP100, in opposition to multiple amino acid substitutions, gave rise to a peptide with enhanced antibacterial activity similar to that reported for RW-BP100 [15] and for other Trp- and Arg-rich antimicrobial peptides [20,39,53]. In these peptides, hydrophobic interactions, hydrogen bond formation and specially cation–π interactions between Arg and aromatic residues, such as Trp, have a decisive role on peptide’s antimicrobial activity. Interestingly, and in contrast with these peptides, our results suggest that cation–π interactions involving Lys residues only, can substantially improve the antibacterial activity of CAMP such as BP100 [15,39,40].

Furthermore, W-BP100 should act by a different MOA from that found for BP100, relying on a two-step process interaction behavior initiated by peptide electrostatic adsorption at the membrane interface and surface charge reduction, followed by peptide translocation across the lipid bilayers, appearing as a sequential aggregation and disaggregation of POPC:POPG (1:1) LUV. 

These findings provide important information for the design of CAMP with a broader antibacterial activity, giving insightsabout the structural properties required to clarify, at a molecular level, the MOA of CAMP against Gram-positive and Gram-negative bacteria. Moreover, these results can add the basis for further studies towards the prediction, rational design and development of novel and efficient short antimicrobial peptides that are ideal drug candidates as they can be synthesized quickly and easily but are harder to design and predict.

## Figures and Tables

**Table 1:** Minimum inhibitory concentration (MIC) and minimum bactericidal concentration (MBC) values for BP100 and W-BP100, against Gram-negative and Gram-positive susceptible reference bacterial species.

Bacteria	MIC µmol dm^−3^ (µg mL^−1^)		MBC µmol dm^−3^ (µg mL^−1^)	
	BP100	W-BP100	BP100	W-BP100
*Escherichia coli* ATCC 25922	1.7 (2.4)	0.75 (1.2)	1.7 (2.4)	0.75 (1.2)
*Pseudomonas aeruginosa* ATCC 27853	1.7 (2.4)	1.5–3.0 (2.4–4.8)	1.7 (2.4)	1.5–3.0 (2.4–4.8)
*Staphylococcus aureus* ATCC 29213	27 (38)	1.5 (2.4)	27 (38)	1.5 (2.4)
*Enterococcus faecalis* ATCC 29212	108–216 (154–307)	3.0 (4.8)	108–216 (154–307)	3.0 (4.8)

**Table 2:** Membrane partition of peptides in anionic and zwitterionic LUV.

Peptide	LUV	$K_{p}\;\times \;10^{3}$ (mol dm^−3^)	$I_{L}/I_{W}$	Fitting Equation
BP100	POPC	n.o.	n.o.	n.o. ^1^
	POPC:POPG (1:1)	16.2 ± 3.7	3.16 ± 0.12	1
W-BP100	POPC	2.4 ± 0.2	2.77 ± 0.06	1
	POPC:POPG (1:1)	16.0 ± 1.8	1.37 ± 0.01	1
		80.6 ± 20.0	-	2 ^2^

^1^ n.o.—not obtained; ^2^ peptide/lipid ratio at membrane saturation conditions (σ) = 0.06, corresponding to ~17 phospholipids per peptide in POPC:POPG (1:1) LUV.

**Table 3:** Stern–Volmer constants (*K*_SV_) determined for the quenching of BP100 and W-BP100 by acrylamide in the absence and presence of LUV, by fitting the experimental data to the Stern–Volmer equation for collisional quenching, Equation (3). The presented values are the Mean ± SD of at least three independent experiments.

Peptide	Medium	$K_{\mathrm{SV}}$ (mol^−1^ dm^3^)
BP100	HEPES buffer	6.60 ± 0.15
	POPC	4.32 ± 0.04
	POPC:POPG (1:1)	1.73 ± 0.07
W-BP100	HEPES buffer	22.35 ± 0.28
	POPC	2.73 ± 0.02
	POPC:POPG (1:1)	2.00 ± 0.09

## Supplementary Materials

The following are available online at https://www.mdpi.com/2077-0375/11/1/48/s1, Table S1: Properties of peptides BP100 and W-BP100; Figure S1: Chromatogram of peptide BP100 acquired by analytical HPLC; Figure S2: ESI-IT MS spectrum of peptide BP100; Figure S3: Chromatogram of peptide W-BP100 acquired by analytical HPLC; Figure S4: ESI-IT MS spectrum of peptide W-BP100; Figure S5: Absorption spectra of peptides BP100 and W-BP100; Figure S6: Fluorescence emission spectra of peptides BP100 and W-BP100; Figure S7: Fluorescence emission spectra of titration of anionic LUV with W-BP100 to evaluate membrane saturation; Figure S8:Fluorescence emission spectra of peptide’s quenching by acrylamide; Figure S9: Intensity-weighted size distribution of anionic LUV in the presence of peptides; Figure S10: Intensity-weighted distribution of zwitterionic LUV in the presence of peptides; Figure S11: Number-weighted distribution of zwitterionic LUV in the presence of peptides.
