# Supplementary material for: How Insertion of a Single Tryptophan in the N-Terminus of a Cecropin A-Melittin Hybrid Peptide Changes Its Antimicrobial and Biophysical Profile
Source: Membranes (Basel). 2021 Jan 12;11(1):48. doi: 10.3390/membranes11010048 (PMC7826622; doi:10.3390/membranes11010048)
Supplement: Supplementary file 1 [file membranes-11-00048-s001.pdf]

membranes

Supplementary Material: How insertion of a single tryptophan in the N-terminus of a cecropin A-melittin hybrid peptide changes its antimicrobial and biophysical profile

Ana Rita Ferreira, Cátia Teixeira, Carla F. Sousa, Lucinda J. Bessa, Paula Gomes, and Paula Gameiro

Table S1. Properties of peptides BP100 and W-BP100.

Peptide[a]	BP100	W-BP100
Sequence[b]	KKLFKKILKYL	WKKLFKKILKYL
No. amino acids	11	12
MW calc. (DA)[c]	1419.97	1606.05
MW found (DA)[c]	1419.80	1606.87
Retention time (min)	11.2	12.4
Purity (%)	97.6	98.0
Net charge[d]	+ 6	+ 6
Hydrophobicity (H)[e]	0.427	0.579
Hydrophobic moment (μH)[e]	0.847	0.964

[a] All peptides were produced as C-terminal amides.
[b] Amino acids residues represented by the single letter code as defined by the IUPAC-IUBMB guidelines on nomenclature and symbolism for amino acids and peptides.
[c] Calculated and experimentally found molecular weight (MW) of peptides.
[d] Total charge from lysine residues and the N-terminal amine at pH 7.4.
e Calculated in the HELIQUEST web server [1].

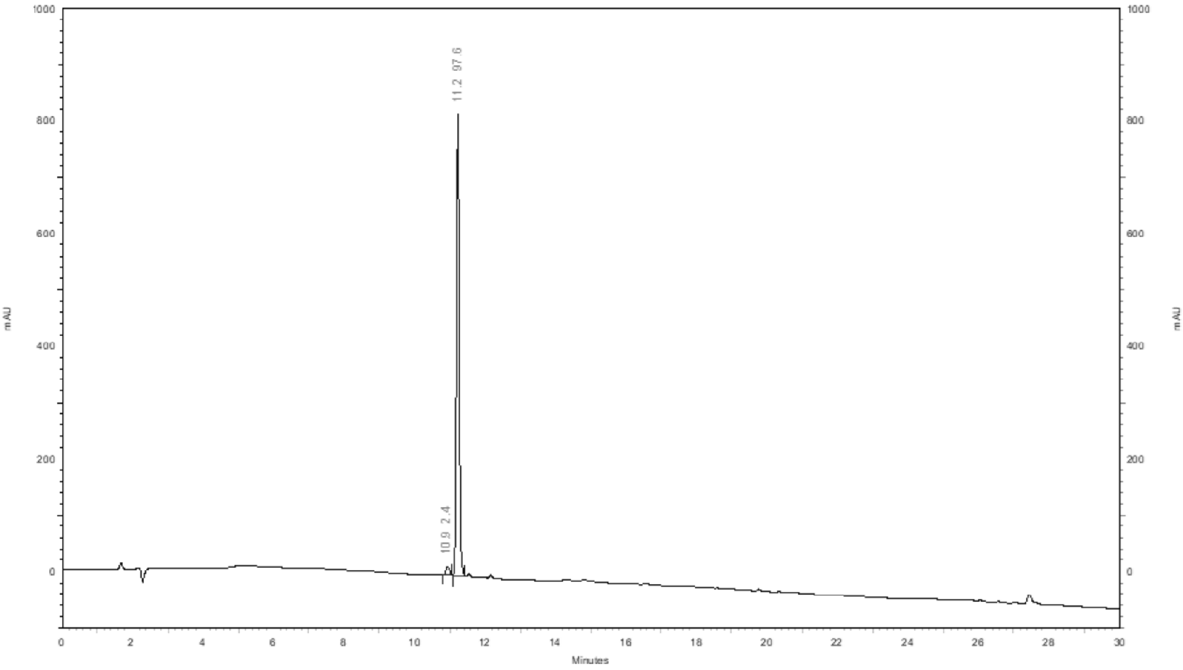

Figure S1. Chromatogram of peptide BP100 acquired by analytical HPLC.

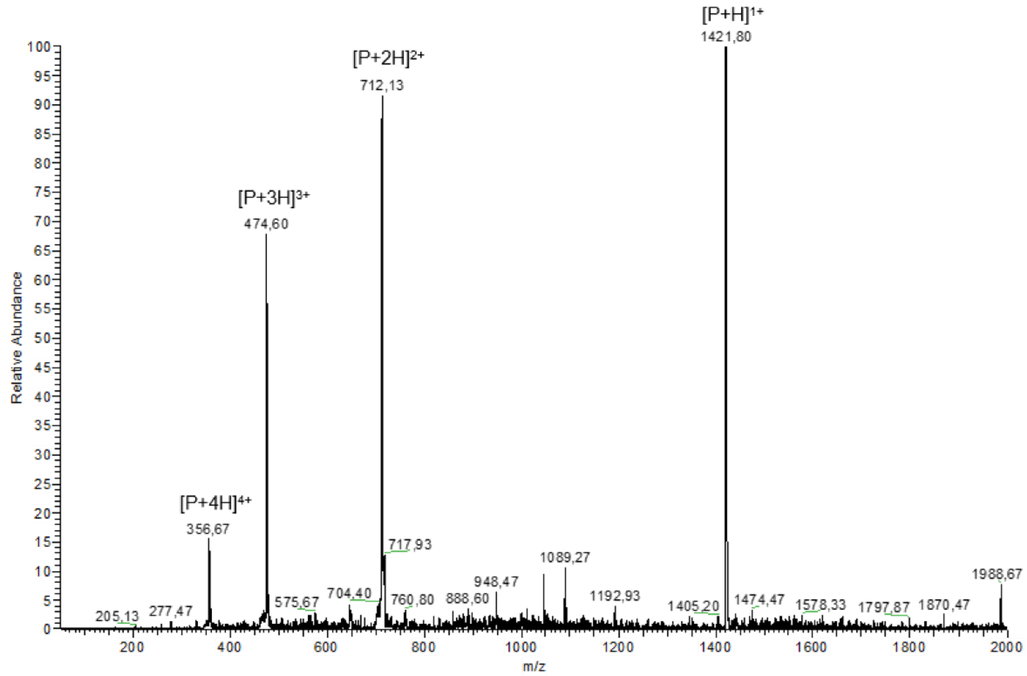

Figure S2. ESI-IT MS spectrum of peptide BP100.

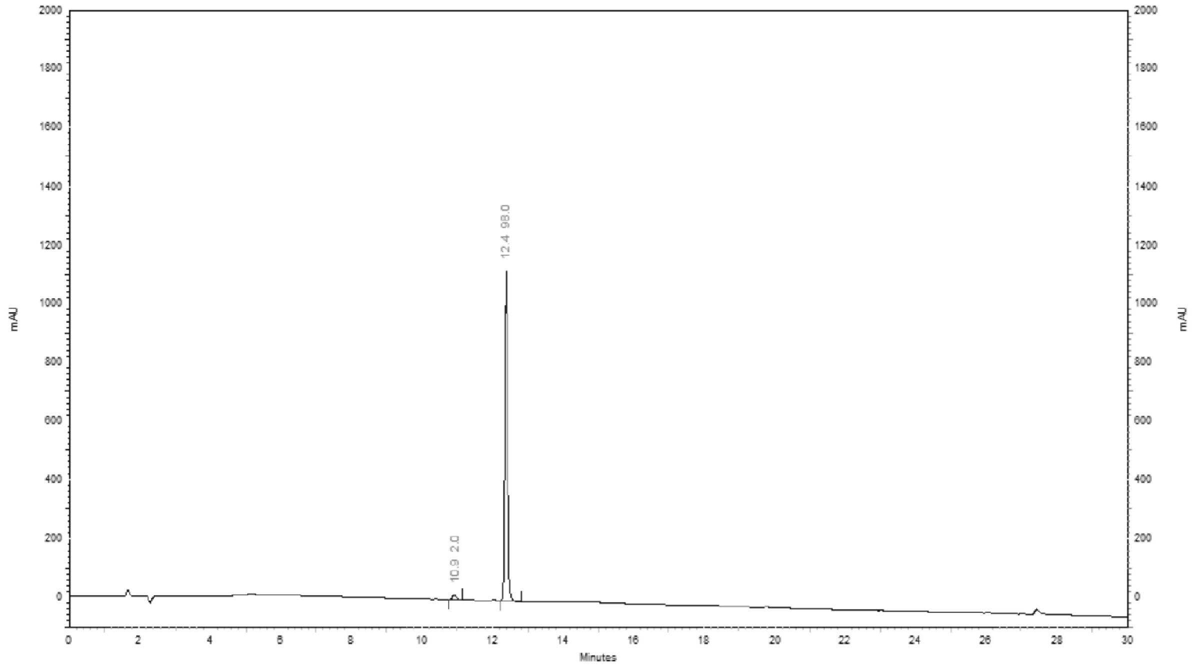

Figure S3. Chromatogram of peptide W-BP100 acquired by analytical HPLC.

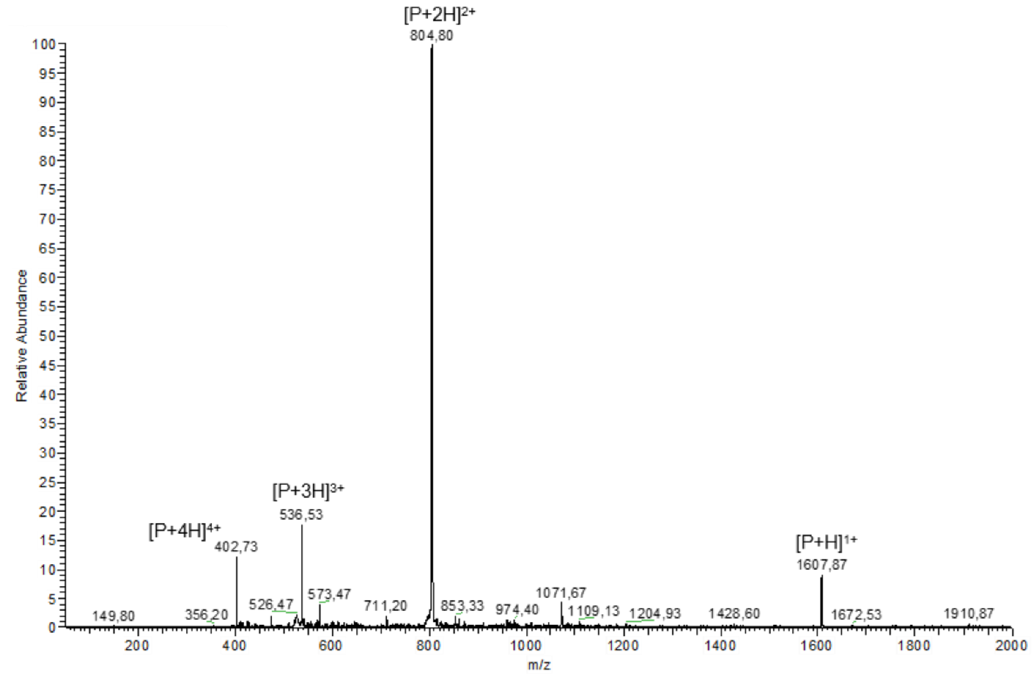

Figure S4. ESI-IT MS spectrum of peptide W-BP100.

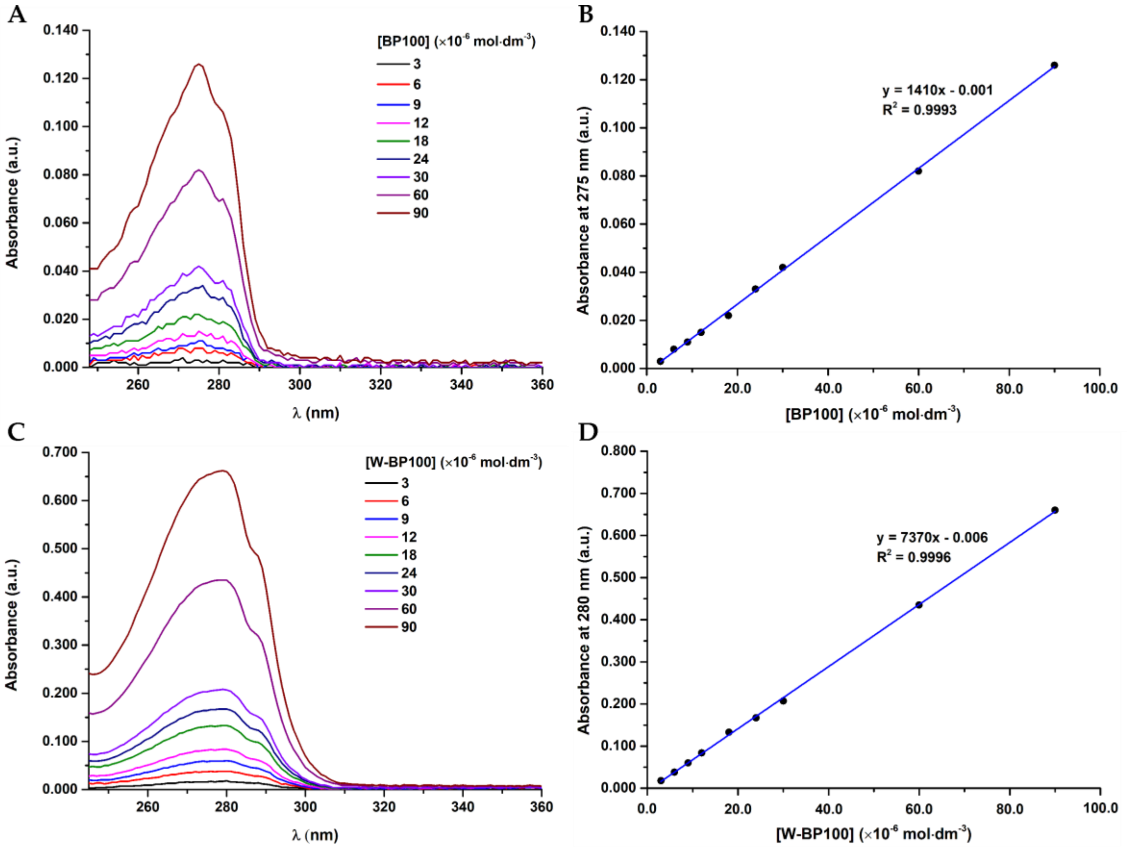

Figure S5. Absorption spectra of peptides BP100 and W-BP100. Representative absorption spectra of increasing concentrations of (**A**) BP100 and (**C**) W-BP100 in aqueous solution (10 mmol dm^{-3} HEPES, 150 mmol dm^{-3} NaCl, pH 7.4), at 25 ± 0.1 °C. Lambert-Beer law of (**B**) BP100 (λ = 275nm) and (**D**) W-BP100 (λ = 280nm).

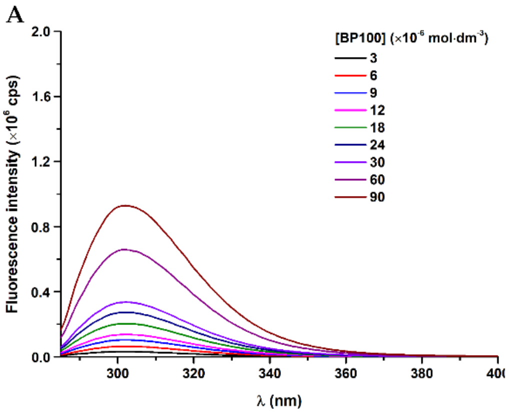

Figure S6. Fluorescence emission spectra of peptides BP100 and W-BP100. Representative fluorescence emission spectra of increasing concentrations of (**A**) BP100 and (**B**) W-BP100 in HEPES buffer (10 mmol dm^{-3} HEPES, 150 mmol dm^{-3} NaCl, pH 7.4), at 25 ± 0.1°C.

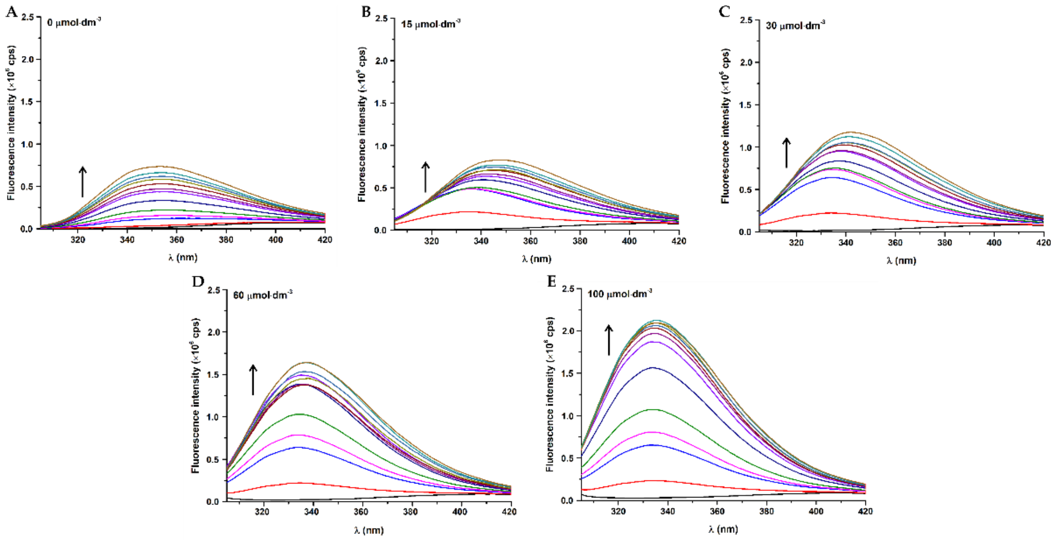

Figure S7. Fluorescence emission spectra of titration of anionic LUV with W-BP100 to evaluate membrane saturation. Representative fluorescence spectra of titration of 0, 15, 30, 60 and 100 μmol dm^{-3} POPC:POPG (1:1) LUV, in the presence of 100 mmol dm^{-3} acrylamide, with increasing concentrations of W-BP100, at 25 ± 0.1 °C. Arrows represent the increase of W-BP100 concentration.

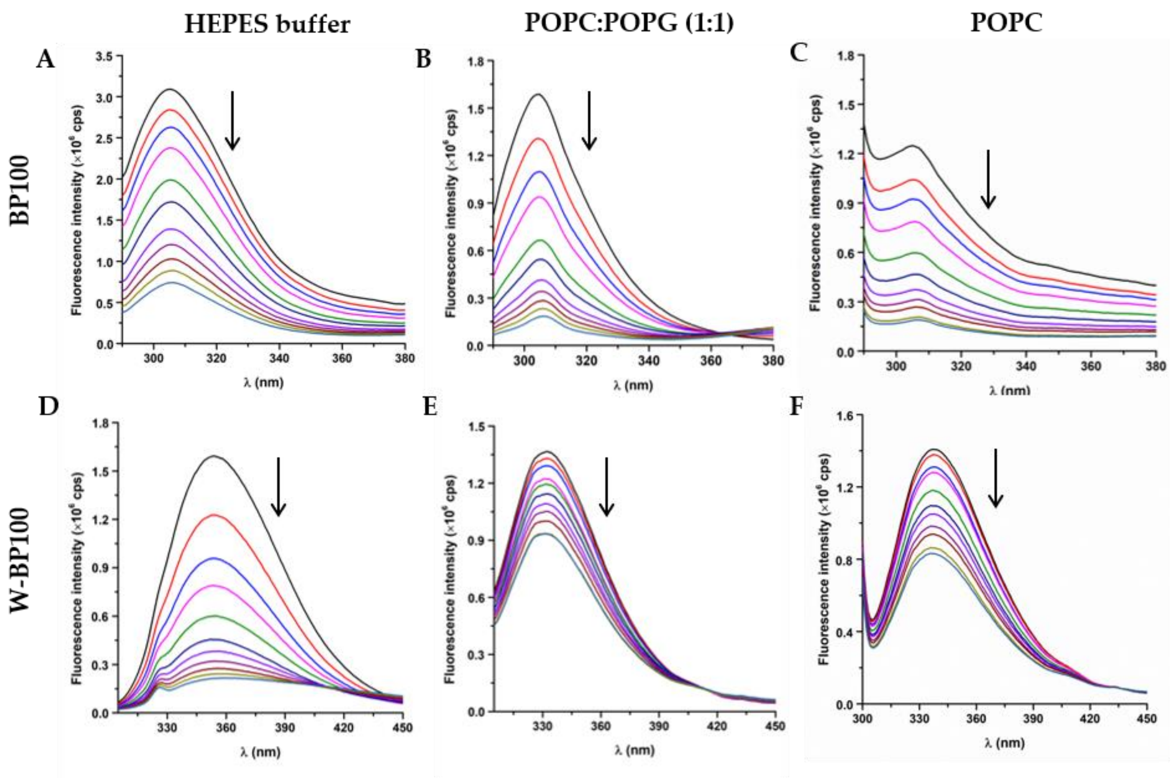

Figure S8. Fluorescence emission spectra of peptide's quenching by acrylamide. Representative fluorescence spectra of 9 µmol dm^{-3} BP100 and W-BP100 in the presence of increasing concentrations of acrylamide in (**A**) HEPES buffer, (**B**) POPC:POPG (1:1) and (**C**) POPC LUV, at 25 ± 0.1 °C. Arrows represent the increase of acrylamide concentration.

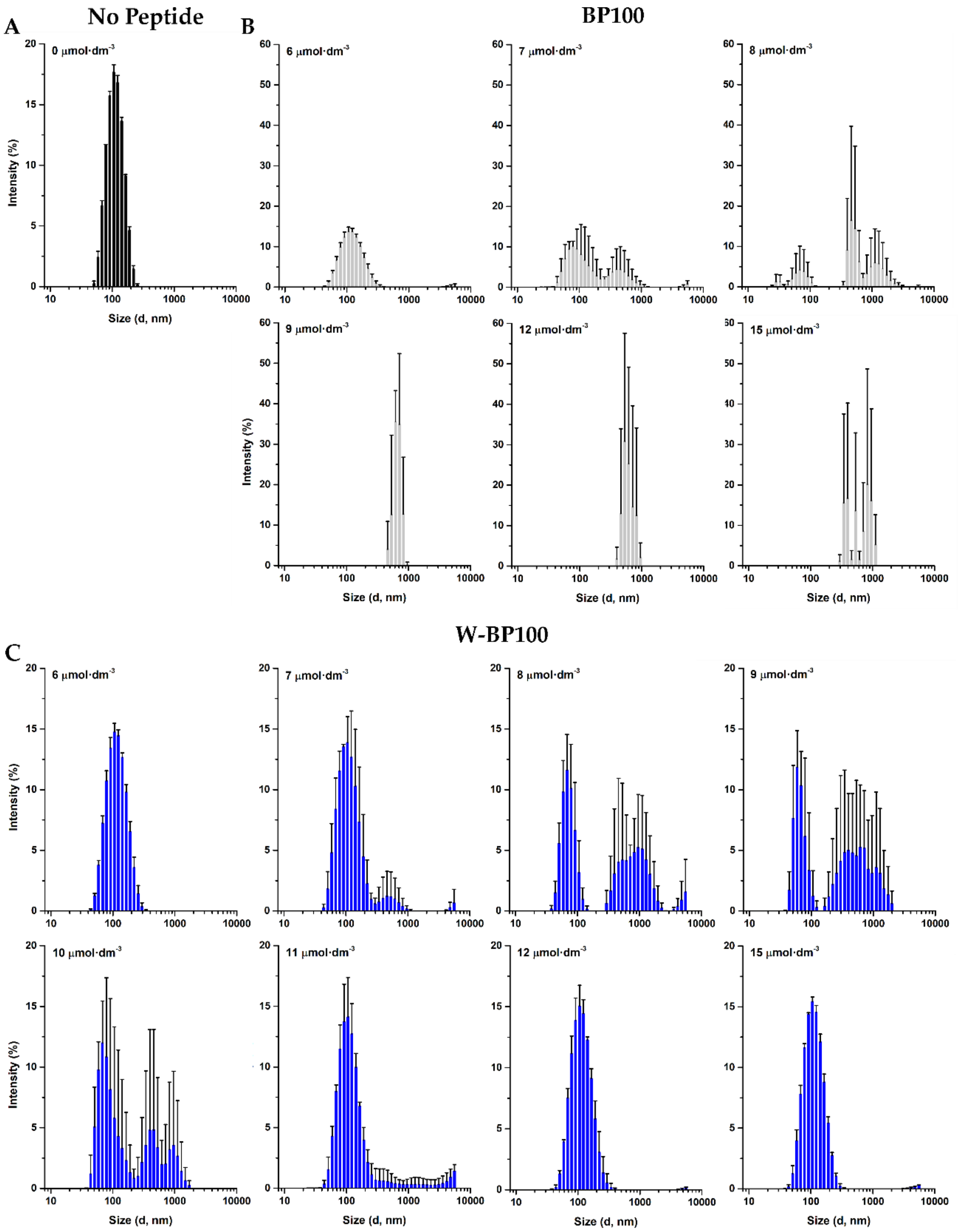

Figure S9. Intensity-weighted size distribution of anionic LUV in the presence of peptides. Intensity-weighted size distribution of 100 µmol dm^{-3} POPC:POPG (1:1) LUV in (**A**) absence and (**B**) presence of 0–15 µmol dm^{-3} BP100 and (**C**) W-BP100. *d* stands for vesicle diameter. Data are the mean ± SD of at least three independent experiments.

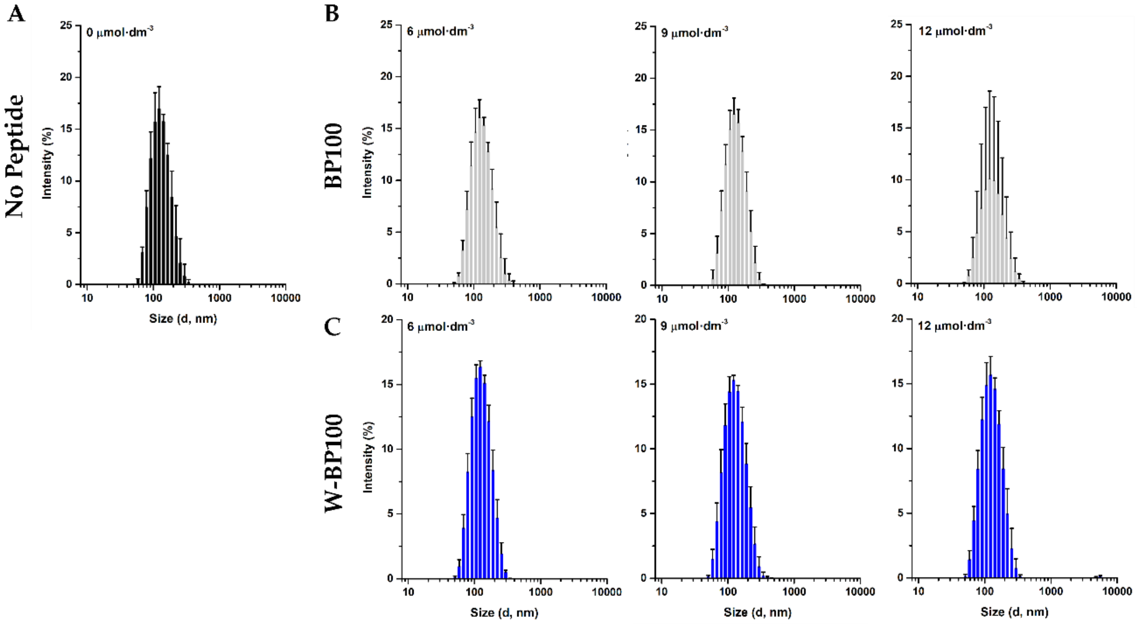

Figure S10. Intensity-weighted distribution of zwitterionic LUV in the presence of peptides. Intensity-weighted size distribution of 100 µmol dm^{-3} POPC LUV in (**A**) absence and (**B**) presence of 0–12 µmol dm^{-3} BP100 and (**C**) W-BP100. *d* stands for vesicle diameter. Data are the mean ± SD of three independent experiments.

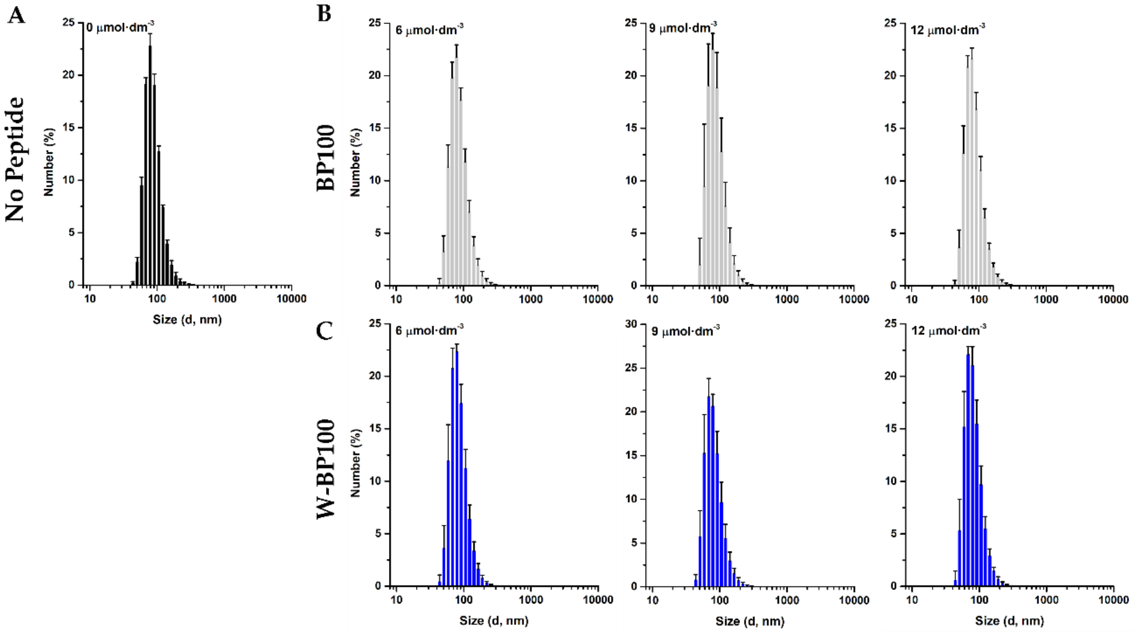

Figure S11. Number-weighted distribution of zwitterionic LUV in the presence of peptides. Number-weighted size distribution of 100 µmol dm^{-3} POPC LUV in (**A**) absence and (**B**) presence of 0–12 µmol dm^{-3} BP100 and (**C**) W-BP100. *d* stands for vesicle diameter. Data are the mean ± SD of three independent experiments.

Reference

1. HELIQUEST. Availabe online: https://heliquest.ipmc.cnrs.fr (accessed on January 25, 2020).
